# The (Non)Impact of Early-Life Job Loss on Later-Life Depressive Symptoms: Citizenship Status and Work Ownership

**DOI:** 10.1093/geroni/igaf122.1446

**Published:** 2025-12-31

**Authors:** Qian Song

**Affiliations:** University of Massachusetts Boston, Boston, Massachusetts, United States

## Abstract

This study examines the impact of non-fault, involuntary job loss experience on depressive symptoms in later life among older Chinese adults. We investigate the heterogeneity of impacts along two important social axes in China – the rural/urban citizenship divide and work ownership in urban China. Utilizing the 2011-2018 China Health and Retirement Longitudinal Study and the 2014 Life History survey, we employed growth curve models with inverse-probability of weighting. Results show that urban citizens experienced heightened depressive symptoms at age 50 (β = 0.75, p <.05), with effects persisting into older age, whereas rural migrants (i.e., rural citizens) showed no significant impacts. Further, only job loss from public sectors adversely affected urban citizens’ mental health in later life (β = 0.91, p <.05), whereas private-sector losses had no impact. Notably, job loss during the 1990s to mid-2000s, the period of critical market transition and massive public-sector layoffs, had the most profound impact on the mental health of older Chinese.

